# Quality of Life Among Adults With Hearing Loss Who Were Prescribed Hearing Aids in Aseer Province, Saudi Arabia: A Cross-Sectional Tertiary Center-Based Study

**DOI:** 10.7759/cureus.45922

**Published:** 2023-09-25

**Authors:** Ahmad M Alrasheed, Montasir Junaid, Khalid T Ardi, Fatma Al-Zahraa M Ebraheem, Omar Z Alaidaroos

**Affiliations:** 1 Otolaryngology - Head and Neck Surgery, Riyadh Third Health Cluster, Riyadh, SAU; 2 Otolaryngology - Head and Neck Surgery, Armed Forces Hospital Southern Region, Khamis Mushait, SAU; 3 Otolaryngology - Head and Neck Surgery, King Faisal Medical City for Southern Region, Abha, SAU; 4 Otolaryngology - Head and Neck Surgery, King Abdulaziz Hospital, Jeddah, SAU

**Keywords:** ioi-ha, hearing aid, aseer region, quality of life (qol), hearing loss, whoqol-bref, hearing-aid

## Abstract

Introduction

Hearing loss affects people of all ages, and it may become a burden for patients as well as for those around them. It leads to social isolation and impacts the quality of life (QOL). Many studies aim to investigate the outcome of hearing aids as an intervention to treat hearing loss. Our study’s objective is to assess the QOL in participants and investigate the possible factors that have an impact on the outcomes of hearing aid use.

Methods

The study adopted a cross-sectional design and was carried out in the Armed Forces Hospital South Region, a tertiary care center in Aseer Province, Saudi Arabia. Participants were patients who presented to the ENT clinic with a complaint of hearing loss from 2017 to 2019 and who were prescribed hearing aids as their treatment. The study uses the World Health Organization Quality of Life BREF (WHOQOL-BREF) questionnaire and the International Outcome Inventory for Hearing Aids (IOI-HA) to measure the QOL and its determinants in patients using hearing aids.

Results

A total of 210 patients were included in the study. Sensorineural hearing loss (SNHL) was found in 72.2%, and 20% of patients were found to have bilateral hearing loss. Moderate or severe hearing loss was found in 80% of the patients in the worst hearing ear. The overall QOL among the participants was satisfactory, with the highest domain score being the social relationship domain (85.9%). The QOL was significantly higher in participants who were in an intimate relationship (P = 0.02). A positive correlation was found between the IOI-HA scores and the WHOQOL-BREF scores in general health (R = 0.14, P = 0.034), psychological health (R = 0.16, P = 0.018), and the overall QOL score (R = 0.15, P = 0.035).

Conclusion

Hearing aids are a cost-effective intervention that improves QOL and prevents associated comorbidities. Compliance and adherence to hearing aids improve the QOL for patients, as well as for their spouses. Patients suffering from hearing loss while also in an intimate relationship had better QOL scores. A correlation was found in outcomes between the IOI-HA scores and the WHOQOL-BREF scores in general health, psychological health, and overall health.

## Introduction

According to the WHO, hearing loss affects approximately 5% of the world’s population and is projected to affect more than 700 million people by 2050 [1]. Worldwide, 430.4 million people are affected with hearing loss that ranges from moderate severity to complete hearing loss [2]. According to the WHO Global Report on Assistive Technology from survey data collected in 29 countries, the prevalence of hearing loss requiring hearing aids ranges from 0.41 to 5.76% (median 1.55%), and the prevalence of access to hearing aids (the ratio of the prevalence of the met need to the prevalence of the need) is 9.9% [3].

Hearing loss can negatively impact a person’s quality of life (QOL), even more so as their better ear worsens [4,5]. Hearing loss was also found to be associated with psychological distress and a decrease in social functioning compared to individuals who do not suffer from hearing impairment [6]. Hearing loss was also reported to be associated with depression, cognitive impairment, and decreasing functional status [7]. Fortunately, the use of hearing aids was found to improve QOL [7,8,9]. Furthermore, it has been suggested that hearing aid use might have a protective effect on cognitive impairment and disability in older patients [7].

The objective of our study was to assess the QOL of patients who visited our hospital with hearing loss from 2017 to 2019 and who were prescribed hearing aids to manage their symptoms. We utilized the World Health Organization Quality of Life-BREF (WHOQOL-BREF) questionnaire as a tool to measure patients’ QOL. Our study also aimed to evaluate the outcome of hearing aid use by using the International Outcome Inventory-Hearing Aids (IOI-HA) tool. The IOI-HA is a questionnaire that was designed to determine, from the patient’s perspective, how beneficial hearing aids are as an intervention for their hearing loss [10]. It comprises seven questions representing different outcome domains [10]. The items, in order, are daily use, benefit, residual activity limitation, satisfaction, residual participation restriction, impact on others, and perceived QOL [10]. Furthermore, we attempted to assess the correlation between the results of these two tools.

## Materials and methods

Study design

The study adopted a cross-sectional analysis of QOL and hearing aid use outcomes using the WHOQOL-BREF and IOI-HA questionnaires. Data were collected via a telephone or in-person interview according to the patient’s preference. Patients with significant difficulty in communication because of their hearing loss were offered an interview through the WhatsApp online chatting platform. A detailed explanation of the questionnaire items was given to patients, and the interview took approximately 30 minutes to perform. Informed consent was obtained from all the patients verbally or through the online chatting platform. Data collected from the patient’s electronic file included their basic demographic data, hearing thresholds from their unaided average pure-tune audiometry results (the average was calculated using 500, 1,000, and 2,000 Hz frequencies), type of hearing loss, and type of hearing aid prescribed to them after the hearing aid fitting process. The questionnaires included questions from the WHOOL-BREF and IOI-HA, as well as questions about general health, comorbidities, and otologic history.

Setting and timeframe of research

Our research was carried out in the Province of Aseer, Saudi Arabia, at the Armed Forces Hospital-Southern Region (AFHSR). The AFHSR is one of the largest tertiary public centers in the province that provides free healthcare to military personnel and their families along with civilians. The hospital serves a region containing a population of four million [11]. Patients were interviewed between the months of August 2021 and May 2022.

Study population

The study population included patients who visited AFHSR with a complaint of hearing loss with or without any other otologic complaints and who were prescribed conventional hearing aids from 2017 to 2019. Only patients between the ages of 18 and 65 were included in the study. A total of 361 patients were contacted and asked to participate, and 210 patients agreed to participate in the study (with a response rate of 58.1%, Table 1).

**Table 1:** Inclusion and Exclusion Criteria

Inclusion Criteria	Exclusion Criteria
Aged 18–65 years old	Aged <18 or >65 years old
Male and female	
Military personnel and civilians	Ineligible patients for AFHSR service
Presented to AFHSR with a complaint of hearing loss	Patients with implantable hearing aids
Diagnosed by an audiologist using pure tone audiometry with conductive, sensorineural, or mixed hearing loss	Patients who were given hearing aids with a tinnitus masker to manage the tinnitus associated with hearing loss
Conventional hearing aids prescribed by an otolaryngologist to manage hearing loss 2017–2019	Patients without a pure tone audiometry result in their electronic file prior to their hearing aid fitting

Statistical analysis

Data were extracted, revised, coded, and fed to the Statistical Product and Service Solutions (SPSS) (version 22; IBM SPSS Statistics for Windows, Armonk, NY). All statistical analyses were performed using two-tailed tests. A P-value of less than 0.05 was considered statistically significant. OOL was assessed using the WHOQOL scale manual, in which scores were converted to a scale from 0 to 10. QOL scores for each domain and overall scores were displayed by the mean, along with the standard deviation. Cross tabulation through one-way analysis of variants (ANOVA) and an independent t-test was used to assess the relationship between QOL, biodemographic data, severity of hearing loss, and type of hearing loss.

The participants’ overall IOI-HA score (out of 35) was displayed as the mean with its standard deviation. Correlation analysis was used to assess the nature and significance of the relationship between WHOQOL-BREF domains and IOI-HA scores. Descriptive analysis based on the frequency and percentage of distribution was done for participants’ biodemographic data, hearing loss data, hearing aid usage, and clinical symptoms.

## Results

Sample description

A total of 210 participants were included in the study, and their ages ranged from 18 to 65 years, with a mean age of 48.8 +/- 13.5 years. The sample contained 115 males (54.8%) and 95 females. Regarding the educational level, 126 (60%) had less than a high school education, while 21 (10%) had a university-level education. When the participants were assessed for comorbidities, 65 patients (31%) had diabetes mellitus, 52 (24.8%) had hypertension, 12 (5.7%) had hypothyroidism, and 112 patients (53.3%) had no comorbidities (Table 2).

**Table 2:** Biodemographic Data of Patients with Hearing Loss Using Hearing Aids Prescribed During Their Visit to the Armed Forces Hospital in the Southern Region 2017–2019

Biodemographic Data	No	%
Age in years		
< 40	50	23.8%
41–59	98	46.7%
60+	62	29.5%
Gender		
Male	115	54.8%
Female	95	45.2%
Relationship status		
Intimate relationship (married)	183	87.1%
No intimate relationship	27	12.9%
Educational level		
Below high school	126	60.0%
High school	63	30.0%
University or graduate level	21	10.0%
Chronic diseases		
None	112	53.3%
Diabetes mellitus	65	31.0%
Hypertension	52	24.8%
Hypothyroidism	12	5.7%
Renal diseases	4	1.9%
Asthma	1	.5%
Depression	5	2.4%
Heart disease	2	1.0%
Others	7	3.3%

Hearing loss was bilateral in 42 patients (20%). Sensorineural hearing loss (SNHL) was found in 72.2% of those with right hearing loss and 73.6% of those with left hearing loss. Moderate hearing loss was found in 34.3% of the participants with right hearing loss and in 36.2% of participants with left hearing loss. Severe hearing loss was found in 27.1% of those with right hearing loss and in 24.8% of those with left hearing loss.

Behind-the-ear hearing aids were prescribed to 75.7% of participants, while 20% were given in-the-ear hearing aids. Hearing aids were used for more than one year by 135 of the participants (Table 3).

**Table 3:** Hearing Loss and Hearing Aid Data of Patients with Hearing Loss Using Hearing Aids Prescribed During Their Visit to the Armed Forces Hospital in the South Region 2017–2019

Hearing Loss		No	%
Right ear hearing loss severity	Normal	16	7.6%
	Mild	27	12.9%
	Moderate	72	34.3%
	Moderately severe	16	7.6%
	Severe	57	27.1%
	Profound	22	10.5%
Right ear hearing loss type	CHL	17	8.8%
	SNHL	140	72.2%
	Mixed	37	19.1%
Left hearing loss severity	Normal	13	6.2%
	Mild	27	12.9%
	Moderate	76	36.2%
	Moderately severe	16	7.6%
	Severe	52	24.8%
	Profound	26	12.4%
Left ear hearing loss type	CHL	16	8.1%
	SNHL	145	73.6%
	Mixed	36	18.3%
Hearing aid usage side	Right	93	44.3%
	Left	75	35.7%
	Bilateral	42	20.0%
Type of hearing aids	Behind-the-ear	159	75.7%
	In-the-ear	42	20.0%
	In-the-canal	6	2.9%
	Completely-in-canal	2	1.0%
Duration of hearing aid usage	< 1 month	6	2.9%
	3 months	13	6.2%
	6 months	23	11.0%
	1 year	33	15.7%
	> 1 year	135	64.3%

Patients were asked about whether they were exposed to acoustic trauma or head trauma. They were also asked about the presence of any otologic symptoms besides their hearing loss, and they were asked whether they were given a treatment alternative to hearing aids if they had undergone any ear surgeries in the past, and how frequently they had visited an otolaryngology or audiology clinic. 

Twenty-four of the participants (11.4%) were exposed to acoustic trauma, while 17 (8.1%) were exposed to head trauma. The most common complaints of patients in the two weeks prior to their interview were recent worsening of hearing and tinnitus (Table 4).

**Table 4:** Risk Factors, Symptoms, and Provided Alternative Treatment Options in Patients with Hearing Loss Using Hearing Aids Prescribed During Their Visit to the Armed Forces Hospital in the Southern Region 2017–2019

	Count	Column N %
Exposed to any of		
Acoustic trauma	24	11.4%
Head trauma	17	8.1%
Both	5	2.4%
None	164	78.1%
Symptoms in the past two weeks		
None	52	24.8%
New worsening of hearing	84	40.0%
Tinnitus	60	28.6%
Vertigo or dizziness	44	21.0%
Wax impaction on HA	37	17.6%
Pus ear discharge	23	11.0%
Were you given alternative treatment to hearing aid?		
Yes	62	29.5%
No	148	70.5%
If an alternative treatment was offered, what type of treatment?		
Nonsurgical	33	53.2%
Surgical	29	46.8%
Undergone ear surgeries		
Yes	27	12.9%
No	183	87.1%
Frequency of visit ENT and audiology clinic		
Never	88	41.9%
Every month	3	1.4%
Every three months	7	3.3%
Every six months	36	17.1%
Every year	76	36.2%

QOL

The overall QOL score among participants was satisfactory at 81.8%, with the highest domain scores in social relationships (85.9%), followed by the environmental domain (83.3%) and psychological health domain (83%, Table 5).

**Table 5:** WHOQOL-BREF Scores of Patients with Hearing Loss Using Hearing Aids Prescribed During Their Visit to the Armed Forces Hospital in the Southern Region 2017–2019

WHOQOL-BREF Domains	Mean	SD
General health	81.3	21.1
Physical health domain	75.5	20.3
Psychological health domain	83.0	16.3
Social relationship domain	85.9	17.9
Environmental domain	83.3	14.6
Overall QOL	81.8	14.7

The overall QOL score was significantly higher among participants in an intimate relationship (82.7%) compared to those who were not in an intimate relationship (75.7%; P-value = 0.022). Other measured factors did not show a significant association with QOL (Table 6).

**Table 6:** WHOQOL-BREF Scores Association with the Bio-Demographic Factors in Patients with Hearing Loss Using Hearing Aids Prescribed During Their Visit to the Armed Forces Hospital in the Southern Region 2017–2019

Biodemographic data	Overall QOL		P-value
	Mean	SD	
Age In years			0.747
< 40	80.4	17.1	
40–59	82.3	14.1	
60+	82.1	13.8	
Gender			0.230
Male	82.9	14.1	
Female	80.4	15.4	
Relationship status			0.022
In relationship	82.7	14.0	
Not In relationship	75.7	18.2	
Educational level			0.235
Below high school	83.0	14.1	
Diploma or high school	80.9	13.8	
University or above	77.4	20.0	
Chronic diseases			0.857
Yes	81.9	14.5	
No	81.7	15.0	

Quality of hearing without hearing aid use was a significant determinant in QOL scores. Participants who faced only slight difficulty in hearing without a hearing aid showed an average score of 86% compared to 76.5% of those who reported that they could not hear at all without using their hearing aid (P-value = 0.026).

Association of QOL and IOI-HA

There was a significant positive correlation between the IOI-HA scores and WHOQOL-BREF scores in the domains of general health (R = 0.14, P = 0.034) and psychological health (R = 0.16, P = 0.018), as well as in overall QOL score (R = 0.15, P = 0.035, Table 7).

**Table 7:** Correlation between WHOQOL-BREF and the International Outcome Inventory-Hearing Aids Scores of Patients with Hearing Loss Using Hearing Aids Prescribed During Their Visit to the Armed Forces Hospital in the Southern Region 2017–2019 R: Pearson correlation coefficient

WHOQOL-BREF Domains	IOI HA score	
	R	P-value
General health	0.14	0.043
Physical health	0.06	0.405
Psychological health	0.16	0.018
Social relationships	0.12	0.082
Environment	0.12	0.078
Overall QOL	0.15	0.035
R: Pearson correlation coefficient		

## Discussion

To date, there are no similar studies that have investigated QOL among patients using hearing aids within the same age group as those in our study.

QOL with hearing aid use

The QOL in our sample was shown to be satisfactory, with a mean overall QOL of 81.8% with the social relationship domain scoring the highest at 85.9%. Vuorialho et al. studied the QOL in the elderly before and after hearing aid fitting using multiple tools. When the Hearing Handicap Inventory for the Elderly (HHIE-S) was used, it showed results suggesting that using hearing aids can reverse subjective social, emotional, and communication dysfunctions [12]. The mean HHIE-S score dropped from 28.7 to 12.7 before and six months after the hearing aid fitting, showing improvement from a significant handicap to a mild handicap [12]. However, when the Euroqol 5-Dimension (EQ-5D) was used as a tool to assess the QOL, it failed to show clear improvement when comparing the before- and after-fitting scores [12].

Lotfi et al. reported an improvement in the HHIE score from approximately 66 to 21 in three months following the hearing aid fitting [13]. However, a generic tool such as the EQ-5D has been reported to lack sensitivity when assessing certain diseases [14]. It showed equivocal results when assessing hearing-related QOL (HRQOL) scores, which led Joore et al. to develop the Audiological Disabilities Preference Index (ADPI) to more accurately evaluate the outcomes of hearing loss [14].

When Stark et al. used the Short Form Health Survey 36 (SF-36), it showed a significant deterioration in the general health subscale without any remarkable changes in the other subscales [15]. Moreover, the study found that there was a significant relationship between the severity of hearing loss and the reduction of the HHIE score from pre- to post-hearing aid fitting [15]. Those with a pure tone average of less than 25 dB had less reduction in their HHIE score compared to those who had a pure tone average of more than 35 dB [15]. It was found that the longer the time of hearing aid use, the greater the reduction in the HHIE score [15]. Those wearing their hearing aids for more than four hours a day had an average reduction of 27.23 compared to a mean of 13.4 for those who used their hearing aids for less than an hour a day [15]. However, when Stark et al. used the SF-36, it showed a significant deterioration in the general health subscale without any remarkable changes in the other subscales [15].

The American Academy of Audiology Task Force on the HRQOL concluded that hearing aid use improved HRQOL by reducing the psychological, social, and emotional effects of SNHL [16].

Intimate relationship and QOL 

We found that participants who were in an intimate relationship had a better QOL score (82.7%) compared to those who were not (75.7%, P-value = 0.022). The effects of hearing loss on patients’ intimate partners have been investigated in multiple studies. Patients who were in an intimate relationship disclosed that their hearing loss had an impact on the QOL of their significant other [15]. The most reported problem for both the patient and their significant other was the need to constantly repeat words to be heard. Other complaints included having to increase the volume on devices such as the TV or radio and feelings of frustration [15].

Brooks et al. also reported that the difficulties experienced by the significant other of a person with hearing loss were correlated with the severity of the hearing loss itself [17]. These difficulties were greatly reduced six months after hearing aid fitting [17].

IOI-HA and WHOQOL-BREF

While the WHOQOL-BREF aims to measure QOL by assessing multiple domains, the IOI-HA reflects the patient’s perspective on the effects of using hearing aids. A positive correlation was found between the IOI-HA scores and the WHOQOL-BREF scores in general health, psychological health, and overall health.

Despite the increasing number of studies showing the benefits of hearing aids, Cox et al. reported that only 23% of the elderly with hearing loss were using their hearing aids [18]. Further studies are recommended to investigate the factors that affect compliance with hearing aids as well as hearing aid accessibility.

Our study is limited by its cross-sectional nature. Other studies showed equivocal results when alternative tools were used to assess the QoL and the outcomes of hearing aid use. However, it was evident that hearing aid use, when accompanied by proper counseling and appropriate fitting, can have a positive impact on the patient and their intimate partner. Further studies are required to evaluate factors that impact hearing aid use and compliance, especially in young and middle-aged adults. Moreover, policymakers should facilitate hearing aid accessibility and coverage by medical insurance considering their cost-effective and positive impact on patients’ QOL and decreased morbidity.

## Conclusions

QOL is significantly affected in patients with hearing loss, and multiple studies suggest that hearing aids are a cost-effective intervention that improves QOL. We conclude that hearing loss creates burdens and difficulties for both the patient and their partner. Therefore, involving the patient’s spouse in the management of hearing loss can be beneficial and might influence compliance with hearing aids.

Furthermore, a significant correlation was found in outcomes between the IOI-HA scores and WHOQOL-BREF scores in general health, psychological health, and overall health. Moreover, policymakers should prioritize the necessary regulations to make hearing aids accessible and affordable to those in need. Doing so may decrease morbidities associated with hearing loss. Further studies are recommended to investigate factors that would help patients with hearing loss become compliant with their prescribed hearing aids, particularly in young-to-middle-aged patients. Health officials should also increase public awareness of preventable causes of hearing loss.
